# Supplementary figures and images for: Homeoprotein OTX1 and OTX2 involvement in rat myenteric neuron adaptation after DNBS-induced colitis
Source: PeerJ. 2020 Feb 13;8:e8442. doi: 10.7717/peerj.8442 (PMC7024580; doi:10.7717/peerj.8442)

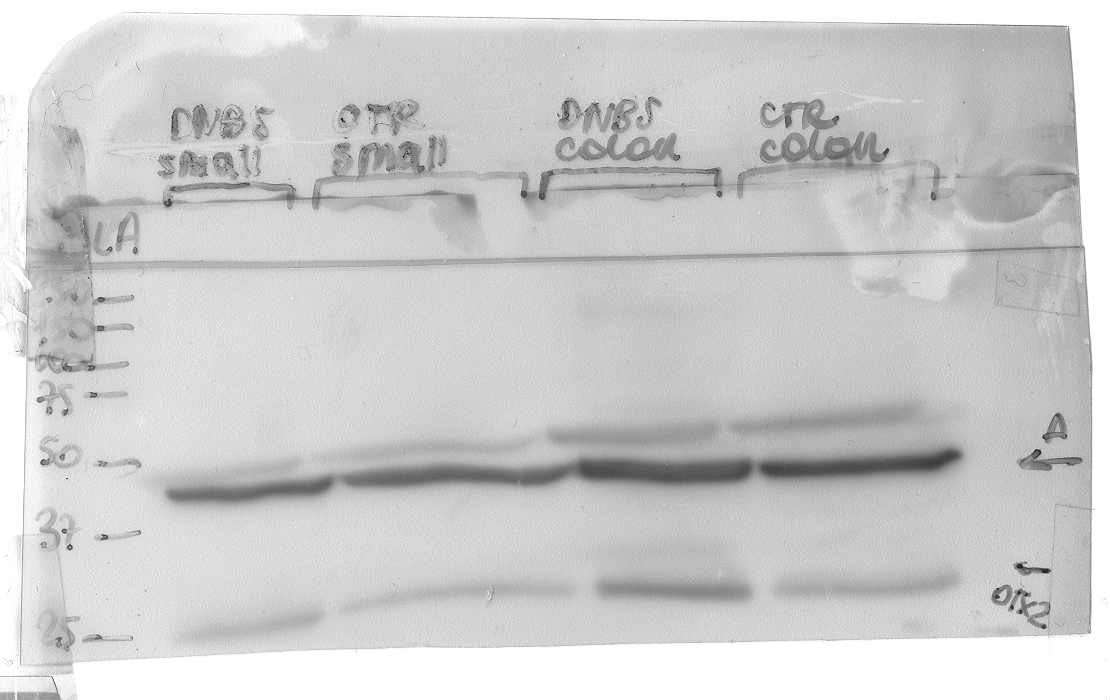

Supplement: Supplemental Information 2 [file peerj-08-8442-s002.png]

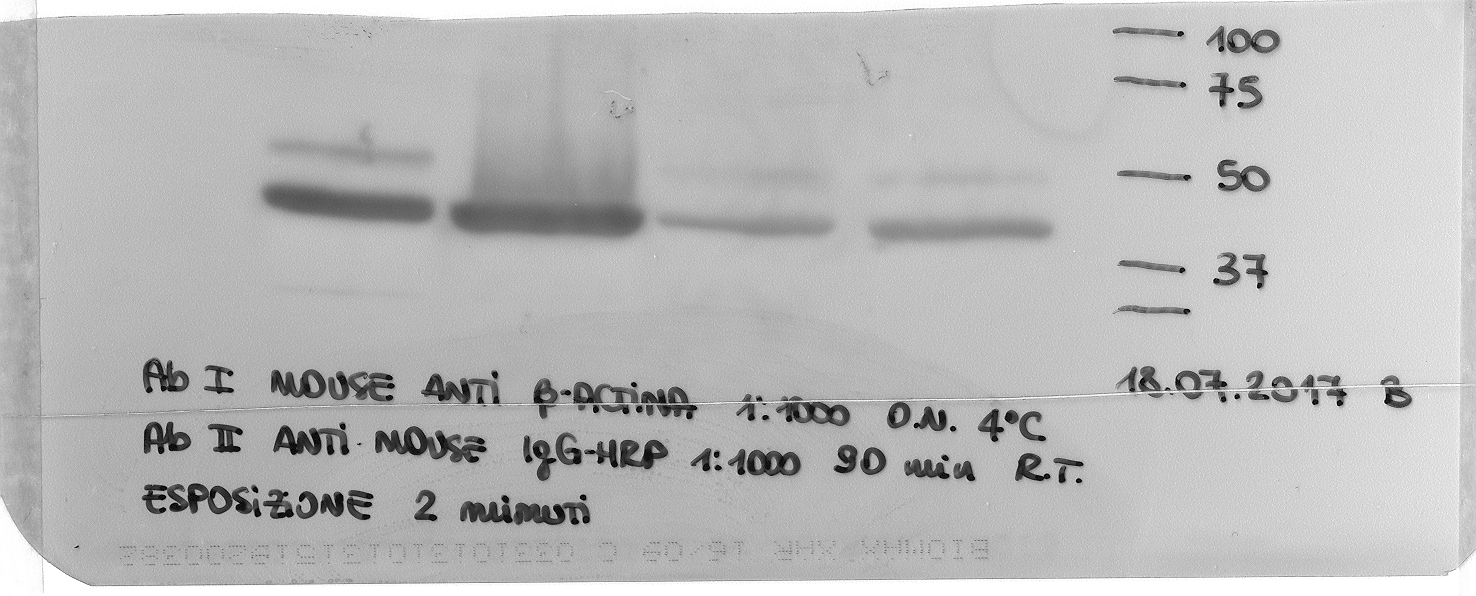

Supplement: Supplemental Information 3 [file peerj-08-8442-s003.png]

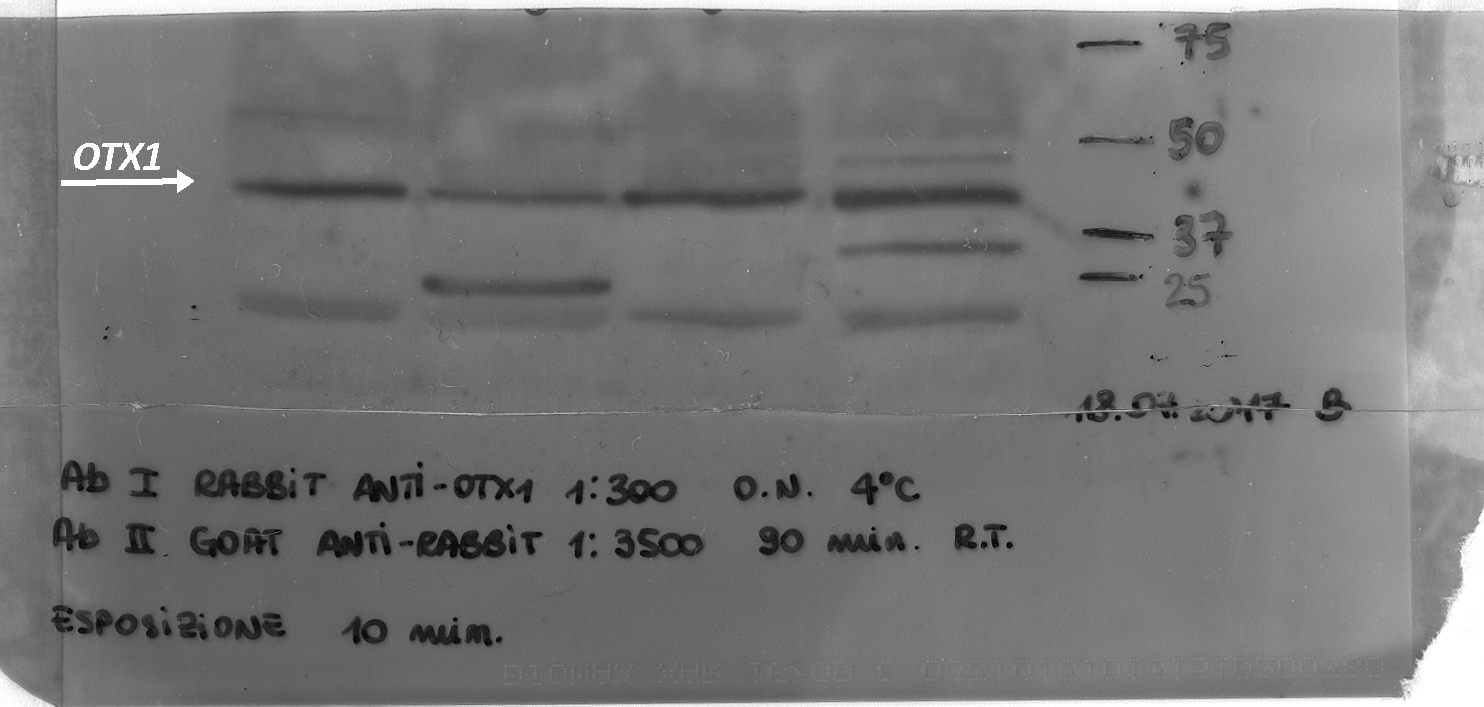

Supplement: Supplemental Information 4 [file peerj-08-8442-s004.png]
